# Rider dislodgement events in U.S. steeplechase racing: participation outcomes in riders and horses

**DOI:** 10.1186/s40621-026-00696-z

**Published:** 2026-06-15

**Authors:** Jeffrey S. Markowitz

**Affiliations:** Independent researcher, Princeton Junction, NJ USA

**Keywords:** Steeplechase, Jump racing, Occupational injury, Falls, Lost rider, Injury surveillance, Participation outcomes, Equine safety

## Abstract

**Background:**

Steeplechase jockeys compete in high-risk races requiring horses and riders to clear fixed obstacles at speed. Although rider dislodgement events are recognized hazards, their incidence and participation consequences are not well characterized.

**Methods:**

This retrospective observational study used publicly available race charts from United States National Steeplechase Association races (2023–2025). Dislodgement events were defined as falls or lost rider events. Incidence rates were calculated per 1,000 starts, and timber and hurdle races were compared using Poisson regression. Rider interruption was defined as ≥ 30 days, with censoring for incomplete follow-up. Horse outcomes were assessed using ≥ 60-day interruption and non-return to jump racing.

**Results:**

Among 3,072 starts involving 83 riders and 710 horses, 187 dislodgement events occurred (60.9 per 1,000 starts; 95% confidence interval (CI), 52.2 to 69.6). Rates were higher in timber than hurdle races (103.5 vs. 51.8 per 1,000 starts; incidence rate ratio 2.00, 95% CI 1.46–2.73). Of 173 evaluable events, 26 (15.0%) were followed by rider interruption. At the rider level, 19 of 44 riders (43.2%) experienced ≥ 30-day interruption, and 7 of 38 (18.4%) did not return to jump racing. Among horses with dislodgement events in 2023–2024, 26 of 111 (23.4%) had no subsequent jump-racing start through 2025, although some continued racing on the flat.

**Conclusions:**

Rider dislodgement events were common and more frequent in timber races. Most riders returned quickly, but a subset experienced interruption or non-return. Participation-based measures may complement traditional injury surveillance in jump racing.

**Supplementary Information:**

The online version contains supplementary material available at 10.1186/s40621-026-00696-z.

## Background

 Horse racing exposes riders to substantial occupational hazards, with falls representing the primary mechanism of traumatic injury in racing [1–2]. Fall rates are higher in jump than flat racing, reflecting the demands of negotiating obstacles [1–3]. Flat racing is conducted at higher sustained speeds without obstacles, producing a different injury profile in which falls are less frequent but may occur at greater velocity. Injury severity may not increase proportionally with fall incidence, potentially reflecting differences in race dynamics, approach speeds, and fall characteristics [1–2].

Following a fall or dislodgement event, riders are typically evaluated by on-site medical personnel and may require clearance to continue riding, although protocols vary. Because riders typically function as independent contractors and income is closely tied to participation, interruption and non-return provide useful indicators of occupational impact.

Steeplechase racing requires horses and riders to clear fixed obstacles in races generally longer than flat races, which may contribute to fatigue. In U.S. steeplechase racing, hurdle and timber races differ in obstacle structure and distance: hurdle races use standardized brush-type “National” fences approximately 52–54 inches high, whereas timber races use more rigid solid obstacles of comparable or slightly greater height. Jump racing occurs internationally at much larger scale, particularly in Great Britain and Ireland, whereas the U.S. circuit is relatively small.

Most jump-racing epidemiology has focused on horses, including risk factors for falls and fatal outcomes [4–7]. Rider-focused studies have emphasized fall incidence and injury patterns [1–2], with less attention to participation outcomes such as return to riding or time lost after dislodgement. As a result, little is known about how dislodgement events affect riders’ ability to continue racing or how these events relate to subsequent horse outcomes.

Falls are also associated with serious outcomes in horses, including fatal musculoskeletal injury, with higher fatality rates reported in jump racing compared with flat racing [4–6].

The present study examines rider dislodgement events in U.S. National Steeplechase Association (NSA) racing, compares incidence between hurdle and timber races, and evaluates participation outcomes for riders and horses. This study was designed as a descriptive analysis of dislodgement events and participation outcomes using race chart data, not as a risk factor analysis. Relevant covariates (e.g., rider characteristics, injury data) are not available in publicly available race chart data.

## Methods

### Study design and data sources

This study examined rider dislodgement events in National Steeplechase Association (NSA) jump racing in the United States between 2023 and 2025. The U.S. steeplechase season runs from March through November. Publicly available race charts (official records summarizing results, finishing positions, and key race events such as horse falls and rider dislodgements) were reviewed for all eligible races during the study period. All races were conducted on turf (grass) surfaces, and horses carried assigned weights determined by race conditions. Riders participating in NSA steeplechase racing are required to hold appropriate licensure and be approved to ride, although specific qualification criteria are not captured in race chart data. Races included a mix of maiden/novice and open/stakes conditions and assigned rider weights reflected standard jump race conditions (approximately 140 to 170 pounds). Rider demographic characteristics, including sex, were not available in race chart data. Race meetings include routine safety oversight by officials, veterinary, and medical personnel; however, specific race-day procedures are not captured in race chart data.

Flat races, training races (non-official races without purses), and Steeplethon races (hybrid flat/jump races) were excluded to maintain comparability with standard hurdle and timber races. Rider status was not recorded; however, because amateur riders primarily compete in excluded training races, the analytic sample largely reflects professional race participation. The final analytic dataset included 3,072 starts recorded by 83 riders and 710 horses.

### Event definitions

Rider dislodgement events were identified from standardized outcome fields in official NSA race charts, where falls (F) and lost rider (LR) events are recorded as part of the order of finish. Events were classified as falls, defined as instances in which the horse fell and the rider was unseated, or lost rider events, defined as instances in which the rider became dislodged without the horse falling. The primary event definition was the combined measure of falls and lost rider events.

### Rider participation outcomes

To evaluate rider participation outcomes, the interval between a dislodgement event and the rider’s next recorded jump-racing start was calculated. Only riders involved in dislodgement events were followed for outcomes. Riders who mounted additional horses later on the same race card were included in the analysis, and the interval was measured from the rider’s final race on that day. This approach accounts for the possibility that injury severity may not be immediately apparent, as riders may initially continue riding before subsequently discontinuing participation.

In U.S. steeplechase racing, there is no fixed limit on the number of mounts a jockey may ride at a given meet; participation is instead constrained by race scheduling and fitness to ride. Accordingly, riders may have multiple mounts on a single race card,

Work interruption was defined as an interval of at least 30 days between the rider’s final race on the day of the event and the next recorded start during the active racing season. Because U.S. steeplechase racing is seasonal (March–November), late-season events may limit opportunities for return within the same calendar year. Modified criteria were applied for November events, and late 2025 events were treated as administratively censored; however, seasonal timing may still influence classification of interruption. A ≥ 30-day interval was used to define meaningful interruption, reflecting the typically frequent opportunities for riders to compete.

Riders with dislodgement events during the 2023–2024 seasons who did not record a subsequent start through the end of the 2025 season were classified as not returning to jump racing. Participation outcomes were based on recorded starts within the U.S. NSA steeplechase circuit; riding activity in other jurisdictions was not captured, and riders classified as not returning may have continued riding outside the U.S. steeplechase circuit.

### Horse outcomes

Horse outcomes were evaluated as secondary endpoints using publicly available race records. Horse-level analyses were restricted to horses involved in rider dislodgement events. Dislodgement events included both falls (horse and rider) and lost rider events (rider unseated without the horse falling). Short-term interruption was defined as an interval of ≥ 60 days between the event and the horse’s next recorded start. A ≥ 60-day interval was used to define substantial participation disruption, recognizing longer intervals between horse starts.

For longer-term outcomes, horses with dislodgement events in 2023–2024 without a subsequent jump-racing start through the end of the 2025 season were classified as not returning. Horses that subsequently competed in flat races were identified during manual review and were not considered to have ceased racing activity, although they were classified as not returning to jump racing.

Horses were further categorized as experiencing immediate cessation if no subsequent race of any type was recorded, or delayed cessation if one additional race was recorded before no further racing occurred through December 31, 2025. Fatal outcomes were described among horses with immediate cessation. Fatal outcomes were not systematically recorded in race charts and were identified through follow-up review of individual horses using publicly available race records and external sources, including cross-referencing with publicly reported information, consistent with previously described verification methods [8]. Fatality verification was performed only for 2024.

For both riders and horses, follow-up was administratively censored on December 31, 2025.

### Statistical analysis

#### Riders

Incidence rates of rider dislodgement events were calculated per 1,000 starts overall and by race type with 95% confidence intervals (CI). Incidence rate ratios comparing timber and hurdle races were estimated using Poisson regression with a log link and 95% CIs. Descriptive statistics were used to summarize rider participation, which was highly skewed; therefore, both median (interquartile range) and mean (standard error) are reported.

Among dislodgement events, rider work interruption was evaluated as the primary outcome. The proportion of events followed by ≥ 30-day interruption was calculated among evaluable events, defined as those with sufficient follow-up to determine participation outcomes, excluding administratively censored events and those without a subsequent recorded start. At the rider level, the proportion of riders experiencing at least one interruption and the proportion not returning to jump racing were also calculated.

#### Horses

Horse-level analyses were secondary and descriptive. The number of horses experiencing one or more dislodgement events and the distribution of events per horse were summarized.

For horses with dislodgement events, time to next start was calculated, and interruption (≥ 60 days) was estimated among events with observable follow-up, with horses lacking a subsequent start treated as right-censored. Longer-term outcomes were summarized as the number and proportion of horses not returning to jump racing. Outcomes were further categorized as immediate cessation, delayed cessation, or continuation in flat racing. Fatal outcomes were reported descriptively.

All analyses were conducted using SAS version 9.4 (SAS Institute Inc., Cary, NC, USA).

### Ethics approval and consent to participate

This study was based on publicly available race records. All data, including horse identifiers, are in the public domain. The study did not involve direct interaction with human participants or access to identifiable personal data; therefore, institutional review board approval and informed consent were not required.

## Results

Counts presented across tables reflect different analytic units (starts, events, riders, and horses), with denominators defined within each table; these units are not directly comparable across analyses.

### Rider dislodgement events and participation outcomes

A total of 3,072 starts involving 83 riders, 424 races, and 710 horses were included in the analysis, including 2,531 in hurdle races and 541 in timber races (Table 1). The number of starts per rider ranged from 1 to 276, reflecting substantial variability in rider exposure. Not all riders participated in both race types: 40 (48.2%) rode in both hurdle and timber races, 31 (37.3%) exclusively in hurdle races, and 12 (14.5%) exclusively in timber races.

Across these starts, 187 rider dislodgement events were identified, corresponding to an overall incidence of 60.9 per 1,000 starts (95% CI 52.5–70.2) (Table 2). Dislodgement events were unevenly distributed across riders: 44 of all 83 riders (53.0%) experienced at least one event, with a median of 3.5 events (IQR 1.0–6.0; range 1–15). Dislodgement event incidence differed substantially by race type. Event rates were higher in timber races (103.5 per 1,000 starts; 95% CI 78.2–134.4) than in hurdle races (51.8 per 1,000 starts; 95% CI 43.3–61.4), with Poisson regression indicating approximately twofold higher incidence in timber races (IRR 2.00, 95% CI 1.46–2.73; *p* < 0.001). In this dataset, hurdle races averaged just over 2 miles in distance, whereas timber races averaged just over 3 miles.

Of the 187 dislodgement events, 173 were evaluable for participation outcomes after applying seasonal criteria. Table 3 summarizes event-level participation outcomes. Of these, 147 events (85.0%) were followed by return to racing within 30 days and 26 (15.0%) by interruption. Among riders with dislodgement events, the median time to next ride was 7 days (IQR 6–14). Eight late-season 2025 events were administratively censored, and six additional events could not be classified due to absence of a subsequent recorded start.

At the rider level, 19 of 44 riders (43.2%) with at least one dislodgement event experienced at least one ≥ 30-day interruption following an event (Table 4). Among riders with events in 2023–2024 and sufficient follow-up (*n* = 38), 7 (18.4%) did not return to jump racing.

### Horse outcomes following dislodgement events

In addition to rider outcomes, horse participation outcomes were also evaluated.

Detailed outcome classification was available for a subset of 51 horses with sufficient follow-up (Table 5). Riders and horses represent separate analytic units, and multiple dislodgement events could occur in the same rider or horse. Of these, 21 (41.2%) were classified as immediate cessation, 16 (31.4%) as delayed cessation, and 14 (27.5%) continued racing on the flat. In 2024, five fatalities were identified among horses with immediate cessation.

For short-term horse outcomes, 132 dislodgement events with a subsequent recorded start were evaluable for participation intervals. Among these, 67 (50.8%) were associated with interruptions ≥ 60 days and 65 (49.2%) returned within 60 days; 55 events were right-censored due to absence of follow-up data (Table 6).

Among horses with dislodgement events in 2023–2024, 26 of 111 (23.4%) did not return to jump racing through the end of the 2025 season; of these, 14 continued racing on the flat (Supplemental Table S1). Among all horses with at least one dislodgement event (*n* = 150), 31 (20.7%) experienced multiple events. Outcome classification was available for a subset of 51 horses with sufficient follow-up, with 21 (41.2%) classified as immediate cessation, 16 (31.4%) as delayed cessation, and 14 (27.5%) as continued racing on the flat.

## Discussion

### Principal findings

This study provides an assessment of dislodgement events and associated participation outcomes in U.S. steeplechase racing. Given the descriptive design, these findings should be interpreted as hypothesis-generating rather than causal. Rider dislodgement events occurred at a rate of approximately 61 per 1,000 starts and were more frequent in timber races than in hurdle races. Most events were not associated with prolonged interruption, with approximately 15% resulting in interruption. These findings indicate common dislodgement events with variable immediate occupational impact.

Differences between hurdle and timber racing in the U.S. include obstacle structure and race distance. International studies have consistently reported higher fall rates in jump racing compared with flat racing [1–3]. The contribution of specific race characteristics was not evaluated in the present study, although prior work has identified race- and horse-level factors associated with fall risk in jumps racing [7]. U.S. flat racing studies have documented lower fall incidence but continued concern regarding jockey injury risk [9]. Similarly, injury surveillance data indicate ongoing concern about horse fatality risk, despite reported declines over time [10]. The present study did not evaluate differences in participation outcomes by race type (hurdle vs. timber), and no conclusions can be drawn regarding factors influencing recovery or return to racing.

### Comparison with prior studies

Cross-study comparisons are challenging because case definitions, racing structure, and data sources vary across jurisdictions. A 5-year Irish study using a similar definition reported 49.5 falls per 1,000 rides [1], modestly lower than the 60.9 per 1,000 starts observed here; however, such comparisons should be interpreted cautiously.

### Rider participation outcomes

The rider participation findings highlight variability in occupational impact, with most riders returning to racing within 30 days, while a subset experienced interruption or non-return. Return to racing, including same-day participation, reflects clearance to continue by on-site medical personnel rather than absence of injury. Same-day return may reflect less severe injury; however, this was not analyzed separately.

Participation-based outcomes represent functional measures that may be influenced by factors beyond injury, including scheduling and economic considerations. Prior studies have typically examined falls and injuries as distinct outcomes, whereas in the present study participation-based outcomes describe functional consequences rather than injury severity. These outcomes reflect participation patterns rather than direct measures of clinical severity.

Rider exposure was highly skewed, with a small number of riders accounting for a large proportion of starts. Among all riders, the median interval between consecutive starts was 7 days (IQR 6–14), whereas for horses it was 35 days (IQR 20–124), reflecting substantial differences in participation patterns.

Missed rides have direct occupational implications for jockeys, as income is tied to participation. Non-return or prolonged interruption may therefore reflect both disruption to participation and other non-medical factors. Serious rider injuries have been described in prior studies [1–3]; however, the outcomes used here do not directly capture injury type or severity. The absence of an unexposed comparison group limits inferences. Occupational dynamics may differ from flat racing, given the smaller pool of riders and potential differences in job security and replaceability.

### Horse outcomes and system-level implications

Interpretation of horse outcomes requires caution. Horse outcomes provide descriptive context for events in which rider dislodgement and horse outcomes co-occur. Among affected horses, a subset did not return to jump racing, including five fatal cases identified in 2024, the only year for which external fatality verification was performed [8]. Dislodgement events may reflect underlying horse injury rather than cause it, and causality cannot be determined; prior work has demonstrated linkage between jockey falls and racehorse injury and fatality outcomes [11]. Non-return does not necessarily indicate adverse outcomes, as horses may transition to flat racing or retire for other reasons. Fatal outcomes were described only among horses involved in dislodgement events (during 2024 only); no population-level fatality incidence rate was calculated.

Prior literature has documented elevated risks of falls and injury in jump racing [1–3], and equine studies have described serious and fatal horse outcomes [4–7]. Recent reporting from the Horseracing Integrity and Safety Authority (HISA) [12] in U.S. flat racing provides broader context, although direct comparison is beyond the scope of the present study. Together, these findings describe patterns of participation rather than definitive measures of injury or welfare outcomes.

### Strengths and contribution to the literature

The present study uses publicly available race chart data to provide a transparent descriptive assessment of dislodgement incidence and participation outcomes for riders and horses. Although limited by the absence of detailed covariate and injury data, this approach allows reproducible assessment across multiple seasons.

### Policy implications

These findings may inform injury surveillance because event counts alone may not capture functional impact. Participation-based measures and transparent public data may support hypothesis-generating evaluation of rider safety and race-type differences.

### Global relevance

Although findings are not directly generalizable beyond U.S. NSA racing, jump racing is conducted internationally at substantial scale, particularly in Great Britain and Ireland, which account for a large proportion of global activity [13–14]. Standardized participation-based measures could support cross-jurisdictional comparisons and improve characterization of the functional consequences of race-day incidents.

### Future research

Future studies in larger or multi-jurisdictional populations should examine rider and horse outcomes by race type, racing environment, and race length. Although the present study includes multiple events per rider, substantial variability in exposure among riders and horses (i.e., number of starts) warrants more formal rate-based approaches. Calculation of rates at the rider and horse level, along with methods that account for repeated events within individuals, may help identify outliers and better characterize heterogeneity in risk. Linkage of race chart data with medical and veterinary records would allow more direct assessment of injury severity, recovery, and the relationship between dislodgement events and subsequent outcomes. Stratified analyses of participation outcomes by race type (e.g., hurdle vs. timber) are needed to determine whether post-event recovery and return to racing differ across racing conditions.

Future work should also evaluate participation-based outcomes in relation to potential risk factors, including rider, horse, and race-level characteristics. In addition, incorporation of standardized measures of participation interruption into injury surveillance systems may help to better characterize the functional impact of race-day events, although further study is needed to determine their validity and applicability across racing contexts. Studies designed to inform evidence-based policy and safety interventions are also warranted.

### Limitations

Several limitations should be considered. The study relied on publicly available race chart data rather than medical or veterinary records, limiting direct assessment of injury severity and reflecting broader variability in injury data collection and reporting across racing jurisdictions [15]. The relatively small size of the U.S. steeplechase circuit constrained statistical power. The descriptive design did not include analysis of risk factors or adjustment for horse-, rider-, or race-level variables; therefore, findings should be interpreted as descriptive rather than causal and do not permit inference regarding determinants of dislodgement events or participation outcomes.

Participation outcomes are influenced by seasonal scheduling and may not fully capture the timing or context of return to racing. Outcomes were also subject to potential misclassification and censoring, as analyses were based on observed activity within the U.S. steeplechase circuit and did not capture participation in other jurisdictions or racing contexts. Non-return to jump racing does not necessarily indicate injury or retirement, as horses may transition to flat racing and riders may pursue opportunities elsewhere.

Classification of horse outcomes was based on race-chart follow-up data; manual follow-up using publicly available records was performed only to verify fatal outcomes in 2024. Outcomes may not capture racing activity beyond the observation period. In addition, variability across riders and racetracks may reflect differences in exposure and small sample sizes. Temporal patterns in dislodgement events across the racing season were not examined and warrant further study.

### Conclusions

Rider dislodgement events are relatively common in U.S. steeplechase racing, with most riders returning quickly but a subset experiencing interruption or non-return. Participation-based outcomes provide a practical but limited measure of functional impact. These findings describe patterns of participation and highlight areas for further study.

**Table 1:** Rider participation and exposure, National Steeplechase Association jump racing, 2023–2025

Measure	Value
Total riders	83
Total starts	3072
Total races	424
Hurdle (fence) starts	2531
Timber starts	541
Median starts per rider (IQR)	8.0 (2.0–34.0)
Range, starts per rider	1–276
Mean starts per rider (SE)	37.0 (7.0)
Rider dislodgement events (F + LR)	187

IQR = interquartile range; SE = standard error; F = fall; LR = lost rider. Starts per rider represent the number of race rides per rider during the study period

**Table 2:** Rider dislodgement events by race type

Race type	Starts	Falls (F)	Lost rider (LR)	Total (F + LR)	Rate per 1000 starts (95% CI)
Hurdle (fence)	2531	74	57	131	51.8 (43.3–61.4)
Timber	541	36	20	56	103.5 (78.2–134.4)
Total	3072	110	77	187	60.9 (52.5–70.2)

Poisson regression: Incidence rate ratio (IRR) for timber versus hurdle = 2.00 (95% CI 1.46–2.73), *p* < 0.001. F = fall (horse fell and rider was unseated); LR = lost rider (rider unseated without the horse falling). Rates are expressed per 1000 starts with exact Poisson 95% confidence intervals (CI)

**Table 3:** Event-level rider participation outcomes following dislodgement events

Outcome	*N*	Percent (95% CI)
All dislodgement events	187	—
Evaluable events^*^	173	100.0
Return within < 30 days	147	85.0 (78.7–89.8)
≥ 30-day interruption	26	15.0 (10.2–21.3)
Administrative censoring (late 2025 events)	8	—
Not classifiable (no subsequent start observed)	6	—

^*^ Evaluable events were those with sufficient follow-up to determine participation outcomes. Percentages are based on evaluable events (*n* = 173). Administrative censoring and non-classifiable events are shown separately and are not included in the evaluable-event denominator. CI = confidence interval

**Table 4:** Rider-level participation outcomes following dislodgement events

Measure	*N*/denominator	Percent (95% CI)
Riders with ≥ 30-day interruption	19/44^*^	43.2 (28.4–59.0)
Riders not returning to jump racing (2023–2024 events)	7/38^†^	18.4 (7.7–34.3)

^*^Denominator includes riders with at least one dislodgement event and sufficient follow-up to assess ≥ 30-day interruption, defined as an interval of ≥ 30 days between the rider’s final race on the day of the event and the next recorded start. ^†^ Denominator includes riders with dislodgement events in 2023–2024 and sufficient follow-up through 2025 to assess non-return to jump racing. CI = confidence interval

**Table 5:** Detailed horse outcome classification following rider dislodgement events

Outcome	*N*	Percent (95% CI)
Immediate cessation of jump racing	21^†^	41.2 (27.6–55.8)
Delayed cessation (after one additional race)	16	31.4 (19.1–45.9)
Continued racing on the flat	14	27.5 (15.9–41.7)
Total evaluated horses	51^*^	100.0

^*^Denominator includes the subset of 51 horses with sufficient follow-up for detailed outcome classification. ^†^Includes 5 fatalities identified in 2024. Percentages are calculated among evaluated horses and reflect participation in jump racing; non-return does not necessarily indicate injury or retirement. Percentages may not sum to 100 due to rounding. CI = confidence interval

**Table 6:** Event-level horse participation outcomes following dislodgement events

Outcome	*N*	Percent (95% CI)^*^
≥ 60-day interruption	67	50.8 (41.9–59.6)
< 60-day return	65	49.2 (40.4–58.1)
Right-censored events	55	—

^*^Percentages are calculated among events with observable follow-up (*n* = 132), defined as events with a subsequent recorded start. CI = confidence interval

## Electronic Supplementary Material

Supplementary Material 1

## Data Availability

No datasets were generated or analysed during the current study.

## Abbreviations

CI: Confidence interval
F: Fall
HISA: Horseracing Integrity and Safety Authority
IQR: Interquartile range
IRR: Incidence rate ratio
LR: Lost rider
NSA: National Steeplechase Association
SAS: Statistical Analysis System
SE: Standard error
