# Supplementary material for: Rider dislodgement events in U.S. steeplechase racing: participation outcomes in riders and horses
Source: Inj Epidemiol. 2026 Jun 15;13:50. doi: 10.1186/s40621-026-00696-z (PMC13292331; doi:10.1186/s40621-026-00696-z)
Supplement: Supplementary file 1 — Supplementary Material 1 [file 40621_2026_696_MOESM1_ESM.docx]

**Supplemental Table S1.** Horses not returning to jump racing following dislodgement events (2023–2024)

| Horse | Meet | Event date |
| --- | --- | --- |
| Bob's Bar | Willowdale | 11-May-2024 |
| By The Riverside | Middleburg | 20-Apr-2024 |
| Cause For Pardon | My Lady’s Manor | 15-Apr-2023 |
| Eryx | Virginia Fall | 12-Oct-2024 |
| Exculpate | Far Hills | 19-Oct-2024 |
| Exuma | Colonial Downs | 12-Aug-2024 |
| Girl Dad | Radnor | 20-May-2023 |
| Go Poke The Bear | Virginia Gold Cup | 4-May-2024 |
| Jimmy Dan | Far Hills | 19-Oct-2024 |
| Johnny Swish | Genesee | 12-Oct-2024 |
| King Bubble | Aiken Spring | 24-Mar-2023 |
| Larger Than Life | Tryon | 13-Apr-2024 |
| Lost Story | Genesee Valley | 14-Oct-2023 |
| Ludicrous Mode | Colonial Downs | 8-Sep-2023 |
| Magic Path | Montpelier | 2-Nov-2024 |
| Market Bubble | Willowdale | 13-May-2023 |
| Pebbly Pour Toi | Far Hills | 19-Oct-2024 |
| Post War | Winterthur | 5-May-2024 |
| Pursuing Pace | Virginia Gold Cup | 4-May-2024 |
| Quality Choice | Genesee Valley | 14-Oct-2023 |
| Secret Soulmate | Tryon | 13-Apr-2024 |
| Tapwood | Virginia Gold Cup | 6-May-2023 |
| To Be Or Not To Be | My Lady’s Manor | 13-Apr-2024 |
| Urban Myth | Aiken Fall | 18-Nov-2023 |
| Variable Cost | Radnor | 18-May-2024 |
| Vincent Van Gogo | Aiken Fall | 18-Nov-2023 |
